# Coupled electro-mechanical response of Bi_2_Te_3_ nanowires probed by electron-beam excitation

**DOI:** 10.1039/d6na00841k

**Published:** 2026-10-26

**Authors:** Abhinav Srivastav, Priyank Tripathi, Reena Yadav, Sahil Verma, Ravita, Mandeep Kaur, Sudhir Husale

**Affiliations:** a Academy of Scientific and Industrial Research (AcSIR) Ghaziabad-201002 India husalesc.nplindia@csir.res.in; b Council of Scientific and Industrial Research, National Physical Laboratory Dr K.S. Krishnan Marg New Delhi – 110012 India; c Department of Physics, Indian Institute of Technology Delhi Hauz Khas New Delhi 110016 India

## Abstract

We investigate the electro-thermo-mechanical response of cantilevered nanowires using *in situ* scanning electron microscopy. Under low beam exposure, thermally driven vibrations manifest as image broadening near the free end, from which Young's moduli of ∼12.56–28.6 GPa are estimated. At higher beam currents, charging-induced forces drive forced oscillations, evidenced by non-Gaussian intensity profiles with enhanced turning-point probability. The spatial amplitude distribution along the nanowire reveals multimode excitation, indicating broadband driving by the electron beam beyond the fundamental mode. Prolonged irradiation additionally induces slow directional bending with an effective charging-relaxation time constant *τ* = 19.96 ± 0.27 s, consistent with a capacitive charging and leakage model. These findings demonstrate electron-beam-induced electro-mechanical coupling in Bi_2_Te_3_ nanowires and establish a contactless approach for probing nanomechanical response and charge-driven dynamics. Although Bi_2_Te_3_ is known to support topological surface states, the present measurements do not directly resolve surface-state transport; a possible contribution of surface states to charge redistribution therefore remains a material-specific interpretation that requires dedicated electronic measurements for confirmation.

## Abstract 2

Electron beam excitation of Bi_2_Te_3_ nanowires for the study of electro-mechanical properties. Analysis is performed by image processing methods.

## Graphical abstract

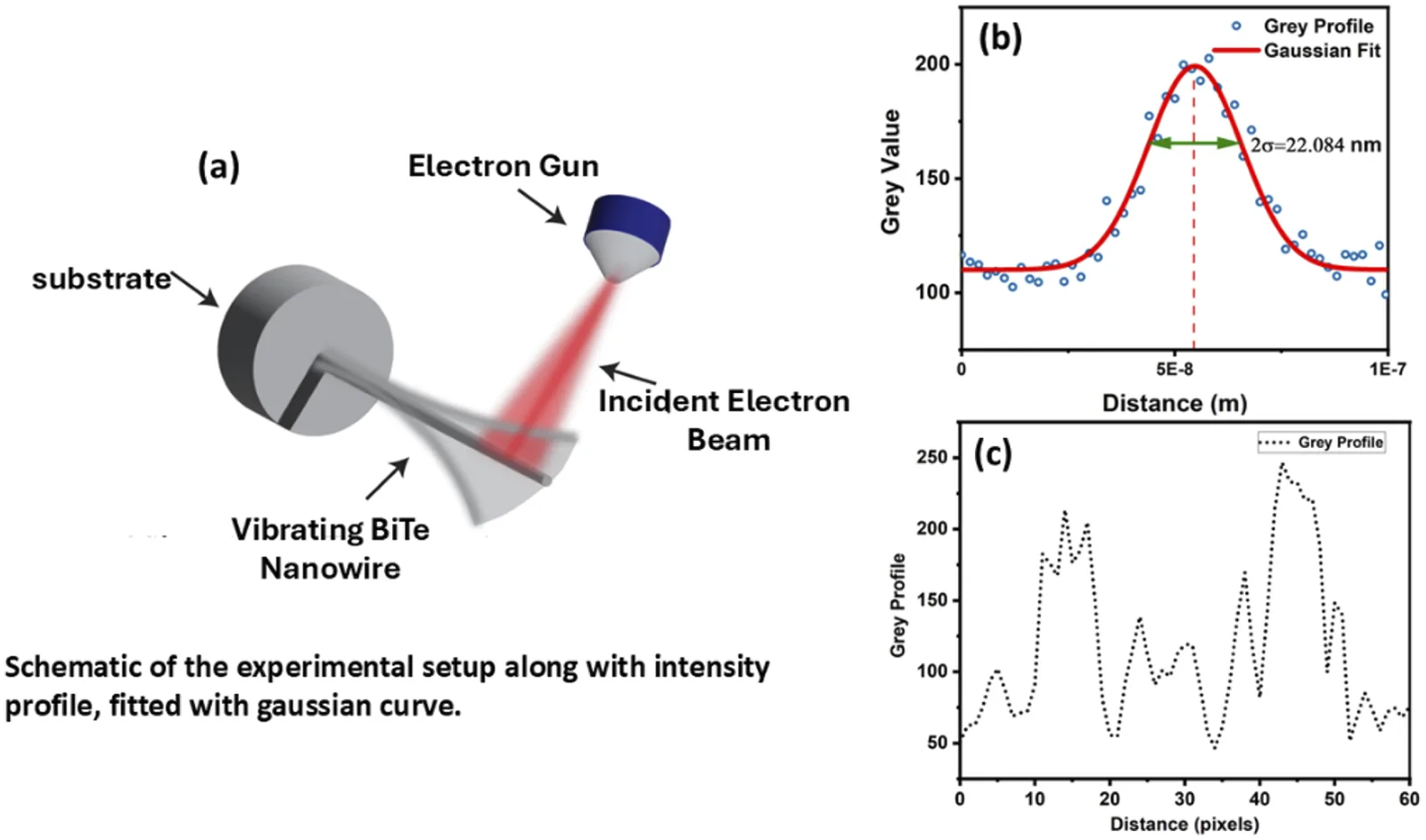

## Introduction

The mechanical response of one-dimensional nanostructures provides a direct route to probe their elastic properties and dynamic behavior at the nanoscale. A clear demonstration of this was given by Treacy, Ebbesen, and Gibson, who showed that thermally driven vibrations of carbon nanotubes can be visualized in a transmission electron microscope, where the apparent broadening of the nanotube image reflects the amplitude of oscillation and allows estimation of Young's modulus approaching the terapascal range.^1–3^ These experiments established that, despite their atomic dimensions, nanowires and nanotubes can be described within the framework of continuum beam mechanics.^4^ Subsequent work has extended these ideas to driven nanomechanical systems, where electrical, electrostatic, or magnetic actuation has been used to excite resonant modes in nanowires and nanotubes, enabling measurements of stiffness, damping, and nonlinear response.^5–7^ In particular, electron-beam-induced excitation inside electron microscopes has emerged as a simple approach where localized charging generates electrostatic forces that can drive oscillations while allowing direct imaging of the motion.^8–10^ More broadly, *in situ* electron microscopy has become an established platform for nanomechanical testing and characterization of crystalline nanowires^11,12^

Most of these studies, however, have focused on materials such as carbon nanotubes, silicon nanowires, and other conventional semiconductors, where the mechanical response is largely independent of the electronic structure. In contrast, topological insulators such as Bi_2_Te_3_ provide a system where electronic and mechanical degrees of freedom may be coupled through surface-dominated transport and charge dynamics.^13,14^ These materials host conducting surface states protected by time-reversal symmetry, characterized by spin-momentum locking. When confined to nanowire geometry, the increased surface-to-volume ratio enhances the role of these states, which can influence charge accumulation, screening, and dissipation processes. At the same time, Bi_2_Te_3_ exhibits moderate stiffness (∼40–60 GPa) and layered crystal structure, making it suitable for forming high-aspect-ratio nanowires that can act as mechanical resonators.^15^ Mechanical properties of Bi_2_Te_3_ nanowires have also previously been investigated using atomistic simulations.^16,17^

Here, we investigate the coupled electro-thermo-mechanical dynamics of cantilevered Bi_2_Te_3_ nanowires (nanowire 1 named as NW1 and nanowire 2 named as NW2) under electron-beam excitation inside a scanning electron microscope. The electron beam serves as both the actuation source and probe, where beam-induced charging generates electrostatic forces that drive the nanowire into oscillation while simultaneously enabling real-time imaging.^18,19^ By analysing the intensity profiles of the nanowires, we extract vibration amplitudes and estimate the Young's modulus using an approach similar to that employed for carbon nanotubes. In addition to thermally driven motion, we observe forced oscillations and excitation of higher-order modes in nanowire 3 (NW3), indicating that the electron beam provides a broadband excitation mechanism. We further examine the role of electron-beam-induced charge accumulation and leakage in governing the time-dependent bending of nanowire 4 (NW4). These measurements provide a contactless approach for probing the mechanical response of Bi_2_Te_3_ nanowires and demonstrate the role of electron-beam-induced electrostatic effects in driving nanoscale mechanical dynamics.

## Experimental methods

Bi_2_Te_3_ nanowires were mechanically exfoliated from bulk crystals and examined inside a FESEM under high-vacuum conditions, where selected cantilevered nanowires exhibited electron-beam-induced oscillatory motion. Vibrational amplitudes were quantified by analyzing grey-scale intensity profiles extracted from SEM images and comparing the Gaussian broadening at the clamped base and free end of the nanowire. The spatial distribution and temporal evolution of Bi_2_Te_3_ nanowire vibrations were analysed from SEM images using edge-detection and image-processing techniques. Local vibration amplitudes along the nanowire length were extracted and compared with analytical cantilever mode shapes to identify contributions from different vibrational modes, while sequential imaging under continuous electron-beam exposure was used to study time-dependent bending and charging-induced effects. Further experimental details, imaging conditions, image-processing methods, analytical modelling and data analysis procedures are provided in the SI.

## Results and analysis

Here, Fig. 1 shows a representative SEM image of a Bi_2_Te_3_ nanowire configured in a cantilever geometry. The nanowire is clamped at one end to the substrate while the other end remains free. The overall geometry is well-defined, with a uniform diameter along the length. Under continuous electron beam exposure, the nanowire exhibits a slight broadening toward the free end, while the base remains sharp. This behaviour is consistent with thermally driven vibrations, where the amplitude increases along the length of a cantilever and reaches a maximum at the tip. A similar observation of thermal motion has been reported earlier for carbon nanotubes.^1^ In the present case, the absence of significant distortion near the clamped end suggests that the boundary condition is well-defined and that the observed broadening is unlikely to arise solely from a static imaging artefact and is consistent with actual mechanical motion. The interpretation is further supported by the systematic base-to-tip increase in profile width, which follows the expected displacement trend of a cantilever rather than a spatially uniform imaging broadening. The measured tip profile is treated as a convolution of the static base profile and the displacement distribution. Under the Gaussian approximation, the corresponding variances are additive, allowing the vibration-related contribution to be estimated from the difference between the tip and base variances.

**Fig. 1:**
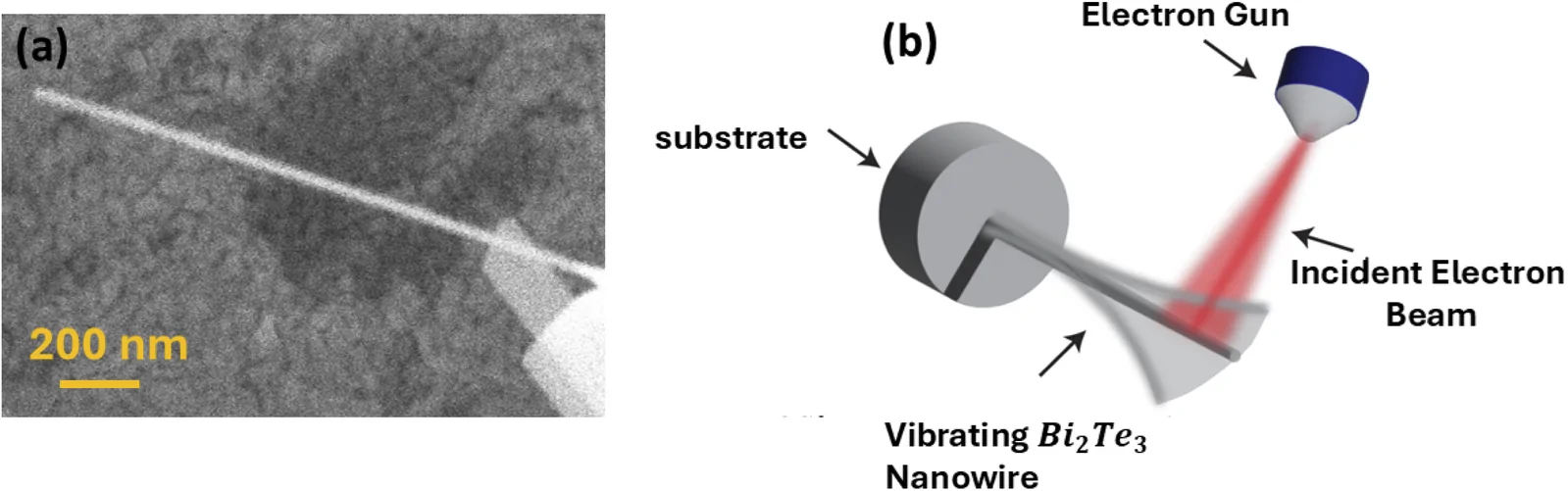
(a) FESEM image of Bi_2_Te_3_ nanowire (NW2) under electron-beam exposure. (b) Representative schematic of vibration of Bi_2_Te_3_ nanowire under electron-beam.

To quantify this effect, grey-scale intensity profiles were extracted perpendicular to the nanowire axis at different positions along its length as shown in. The profile at the base Fig. 2(a) shows a narrow distribution corresponding to the intrinsic imaging resolution, while the profile near the tip is noticeably broader. Both profiles were fitted with Gaussian functions Fig. 2(b), this behaviour is consistent with the expectation that the displacement of a thermally driven cantilever follows a probabilistic distribution arising from stochastic excitation and the increase in variance at the tip is attributed to vibrational motion. This approach assumes that the measured intensity profile is a convolution of the static nanowire image and the probability distribution of its displacement. The difference between the widths of the tip and base profiles therefore provides a measure of the vibration amplitude and the observed increase in broadening toward the free end follows the expected behaviour for a cantilever undergoing flexural vibrations, where the displacement is maximum at the tip. For small-amplitude vibrations, the nanowire acts as an elastic beam governed by the Euler–Bernoulli equation,^20,21^ where the displacement is dominated by flexural modes. Under thermal equilibrium, each vibrational mode carries an average energy of 
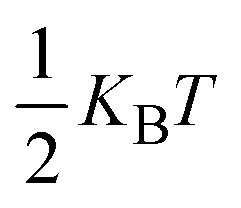
 as given by the equipartition theorem.^22^ This leads to a Gaussian distribution of displacement for each mode, and in the absence of strong external driving, the fundamental mode contributes most significantly to the motion. As a result, the experimentally observed intensity profile near the tip can be approximated by a Gaussian whose standard deviation reflects the root-mean-square displacement of the nanowire tip.

**Fig. 2:**
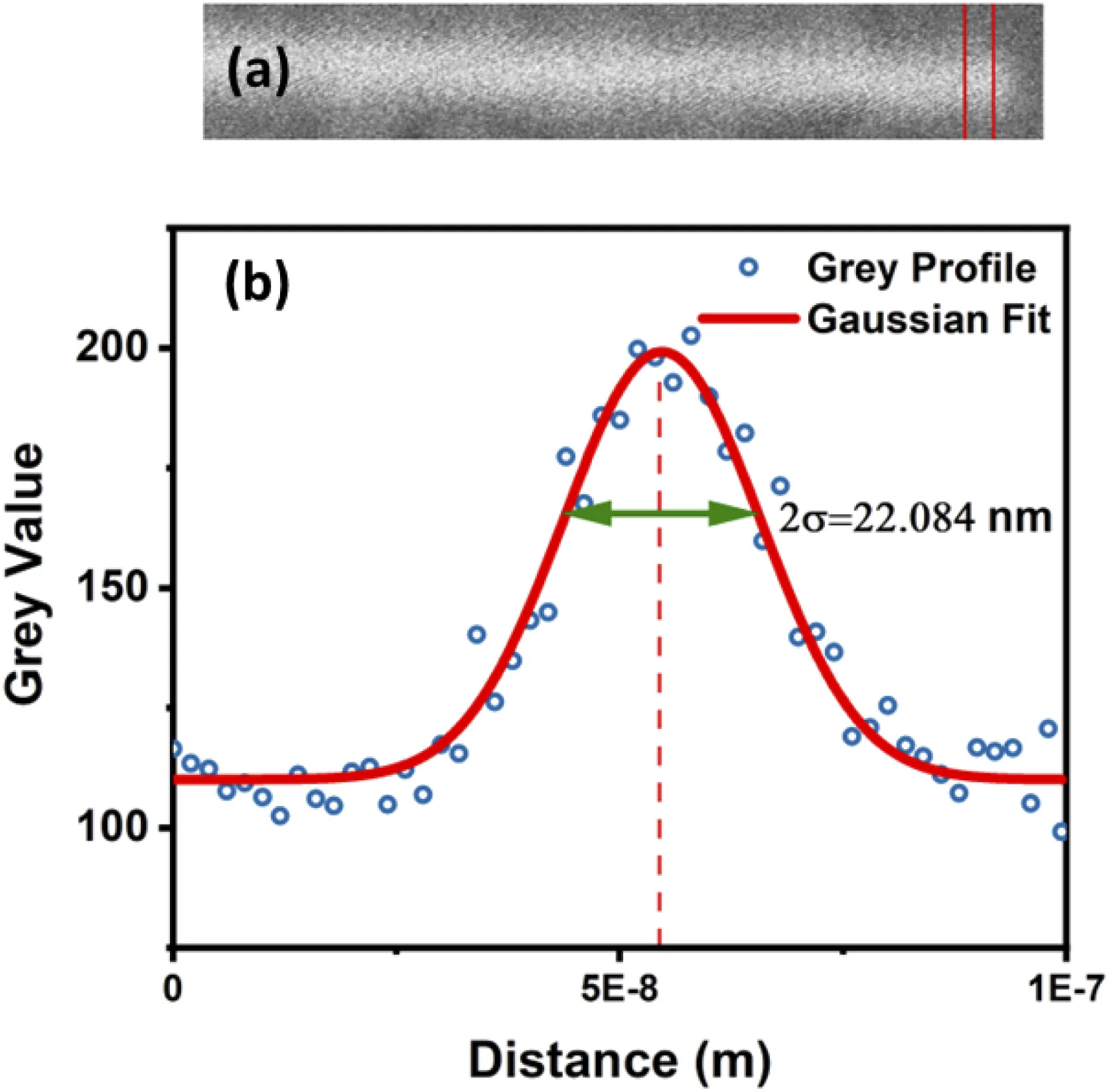
(a) Plot of the intensity distribution along the cross section of the nanowire (NW2), near the tip. (b) The intensity is taken as grey value from the SEM Image which is fitted to a Gaussian curve.

From the Gaussian fit in Fig. 2(b), the standard deviation of the profile is extracted to be on the order of tens of nanometres. However, this value includes contributions not only from vibrational motion but also from the intrinsic imaging resolution and static broadening of the nanowire. To isolate the vibrational component, the measured profile must be considered as a convolution of two Gaussian distributions: one corresponding to the static nanowire (base profile) and the other arising from dynamic motion. In this case, the effective vibrational variance is given by

1 *σ*_vib_^2^ = *σ*_tip_^2^ − *σ*_base_^2^

which allows estimation of the vibration-related displacement contribution relative to the static broadening represented by the base profile.

Using this approach, the extracted vibrational amplitude can be related to the mechanical properties of the nanowire. For a cantilever of length *L*, the mean-square displacement at the tip is linked to the Young's modulus *Y* through the relation derived from beam theory and thermal energy considerations. An initial estimate based directly on the tip profile yields a lower value of the modulus, indicating that neglecting the base contribution leads to an underestimation of stiffness. Once the intrinsic broadening is accounted for, the corrected displacement values provide Young's modulus in the expected range for Bi_2_Te_3_ nanowires.

The agreement of the experimental profile with a Gaussian distribution and its consistency with thermal vibration models indicate that the motion observed in Fig. 2 is predominantly thermally driven, with negligible contribution from higher-order modes at this stage. The absence of asymmetry or multi-peaked features further supports the dominance of the fundamental mode. These results validate the use of SEM-based intensity profiling as a quantitative tool for extracting vibrational amplitudes and mechanical parameters in nanoscale cantilevers.

Fig. 2 shows the grey-scale intensity profile extracted across the nanowire near the free end. The experimental profile is well fitted by a Gaussian, which is consistent with the expected displacement statistics of a thermally driven cantilever. From the beam theory formulation, the transverse displacement of the nanowire is governed by the Euler–Bernoulli equation, and the motion can be expressed as a superposition of eigenmodes with mode shapes *Y*_n_(*x*) satisfying the characteristic equation:^5^2
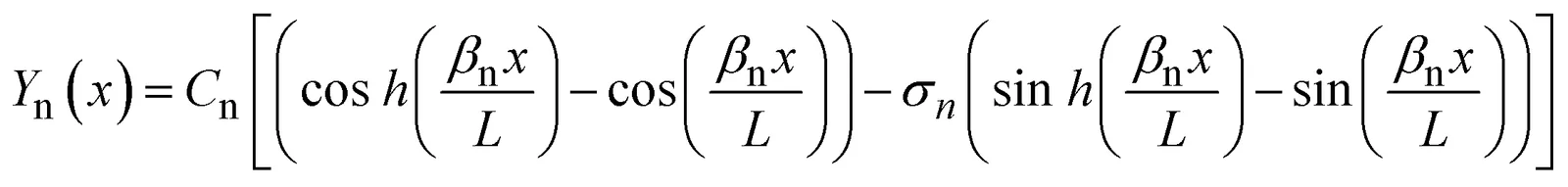
where *β*_n_ are the solutions of the characteristic equation and *σ*_n_ is mode dependent constant.

3 cos (*β*_n_)cos *h*(*β*_n_) = −1

with *β*_1_ corresponding to the fundamental mode (*n* = 1).

Under thermal equilibrium, each mode carries an average energy of *k*_B_*T*(equipartition theorem), which leads to a Gaussian probability distribution of displacement. As the fundamental mode requires the least energy, the motion at the tip is dominated by this mode, and the measured intensity profile can be approximated as

4 *I*(*y*) = e^(−*y*^2^/2*σ*^2^)^

where *σ* represents the root-mean-square displacement of the nanowire tip.

Fig. 3(a) is the marked (base in red and blue in tip) FESEM image of nanowire and Fig. 3 compares the intensity profiles at the free end of the nanowire (Fig. 3(b)) and the clamped base (Fig. 3(c)). The base profile is narrow and represents the static image of the nanowire, while the tip profile is broader due to vibrational motion. To account for this, the observed profile at the tip can be written as a convolution of the static profile *I*_0_(*y*) and a Gaussian distribution *G*(*y*) representing vibration:5
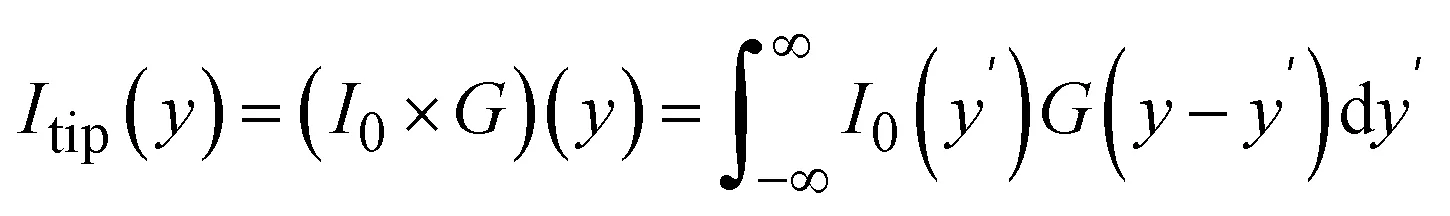
Since both *I*_0_(*y*) and *G*(*y*) are Gaussian, the resulting profile is also Gaussian, and the variances add. This leads to

6a *σ*_tip_^2^ = *σ*_base_^2^ + *σ*_vib_^2^

or

6b *σ*_vib_^2^ = *σ*_tip_^2^ − *σ*_base_^2^

**Fig. 3:**
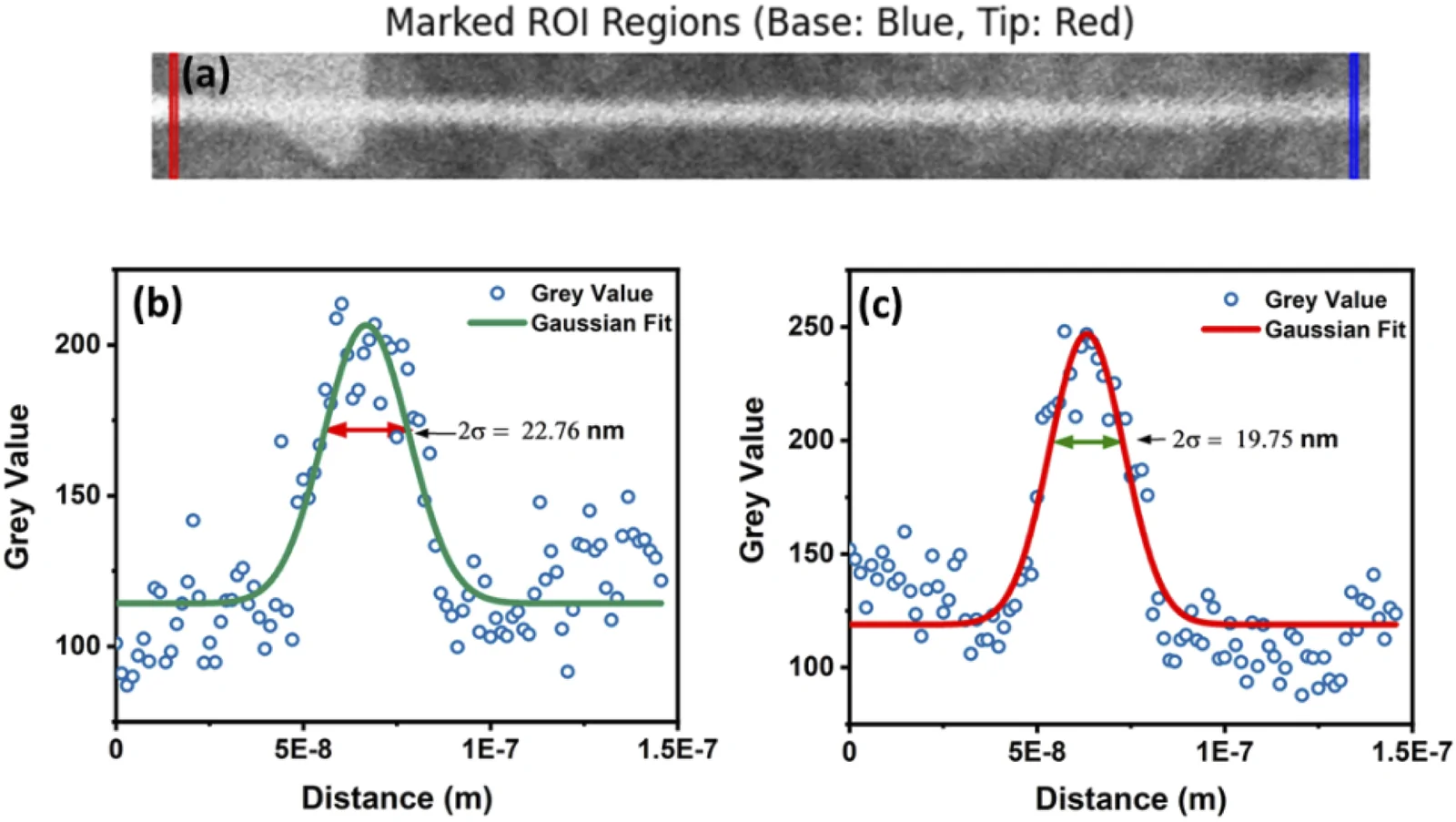
Comparison of intensity profiles at the clamped base and free tip of the nanowire (NW2), (a) FESEM representation of base and tip region of nanowire. (b and c) Gaussian fits to both profiles reveal increased broadening at the tip due to vibrational motion.

Using the relation 6(b), the true vibrational amplitude is extracted by removing the contribution of the static profile. The corrected displacement values are smaller than those obtained from the raw tip profile and lead to Young's modulus values in the range of ∼12.56–22.6 GPa (Table 1), consistent with reported values for Bi_2_Te_3_ nanostructures and well below than single crystal and bulk limit as stated in SI table S1.

**Table 1:** Parametric values obtained from the Gaussian-profile analysis for NW1 and NW2, including nanowire dimensions, profile widths, estimated vibration amplitude, Young's modulus, and spring constant

Parameter	NW1	NW 2
Length, *L* (nm)	∼5300	∼1480
Radius, *R* (nm)	∼23.5	∼10.9
Base profile, *σ*_base_ (nm)	23.52	9.88
Tip profile, *σ*_tip_ (nm)	24.31	11.38
Estimated vibration, *σ*_vib_ (nm)	6.15	5.64
Estimated Young's modulus (GPa)	22.56	12.56
Spring constant (N m^−1^)	1.09 × 10^−4^	1.29 × 10^−4^

Care was taken to minimize and assess possible SEM-related imaging artifacts. The measurements were performed using a high-resolution ZEISS Auriga FESEM, with spatial resolution down to approximately 1 nm and detector capability for image acquisition on the millisecond timescale used in the present measurements. Prior to imaging, the system was calibrated using a traceable calibration sample, and careful fine focusing and beam-wobbling procedures were employed to minimize astigmatism. A large number of nanowires with different geometries, including substrate-supported, standing, and one-end-clamped/suspended nanowires, were examined under comparable imaging conditions. The observed vibrational response was predominantly associated with the mechanically compliant one-end-clamped/suspended nanowires and was not observed in all nanowires within the same imaging region, reducing the likelihood of a common scan-related or charging-induced imaging artifact. During image acquisition, the clamped/base region remained essentially stationary, while the displacement increased toward the free end, which is inconsistent with global stage drift and is consistent with cantilever mechanical motion. Furthermore, the finite imaging broadening was accounted for by using the nanowire base as an internal reference. The vibration-related contribution was obtained from7
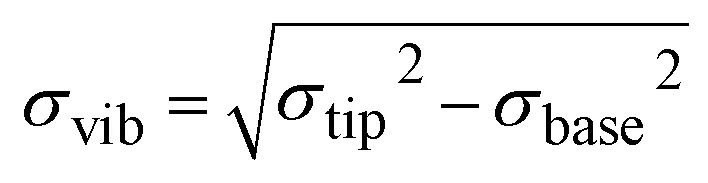
rather than directly assigning the measured tip width to mechanical vibration. Eqn (6) is further used to distinguish mechanical motion from instrumental broadening, the nanowire base was used as a static reference. The measured tip profile was compared with the base profile, and the contribution of static imaging broadening was removed using eqn (7).

Statistical analysis of length scales (length and diameter) is presented in SI Fig. S1, confirming the consistency of reported mechanical parameters. As well as the statistical length scale data used to calculate total uncertainty budget.

The uncertainty in the vibration-related displacement is propagated from the fitted uncertainties of the tip and base Gaussian widths. Contributions from Gaussian fitting, pixel-scale resolution, image noise, and edge-detection uncertainty are incorporated into the corresponding profile-width and geometrical uncertainties. Instrumental broadening that is not independently resolved is included in the static/base-profile contribution. The extracted vibration-related displacement therefore depends on the uncertainties in both the fitted tip and base profile widths, which are propagated to the mechanical parameters obtained from the corrected displacement.^23^ Detailed uncertainty analysis is incorporated in SI, Table S2 demonstrates the measured image processing parameters with uncertainty. Table S3 demonstrates total uncertainty budget along with individual parametric uncertainties calculated using eqn S1–S3.

The comparison between base and tip profiles provides evidence that the observed broadening is not attributable solely to uniform static imaging broadening and is consistent with spatially varying mechanical motion. The symmetric Gaussian shape and monotonic increase in variance toward the tip are consistent with the fundamental mode shape of a cantilever, where the displacement increases along the length and reaches a maximum at the free end. To further assess whether the observed edge broadening could arise from SEM imaging-related effects, control measurements were performed under identical electron-beam and imaging conditions using nanowires that did not exhibit observable mechanical motion.

Control experiments were also performed under identical SEM operating conditions using nanowires that did not exhibit observable mechanical motion. The reference nanowire regions marked by yellow boxes remain essentially unchanged throughout the 0–10 s irradiation sequence, whereas the mechanically compliant nanowire marked by the red box exhibits progressive edge displacement/broadening toward its freely suspended end Fig. 4(a–f). No comparable broadening was observed in the non-vibrating reference nanowires under the same imaging conditions. Together with the stationary clamped region and the spatially localized increase in displacement toward the free end, these observations provide additional experimental evidence supporting the interpretation that the observed edge broadening primarily arises from electron-beam-induced mechanical motion rather than from a common SEM imaging artifact.

**Fig. 4:**
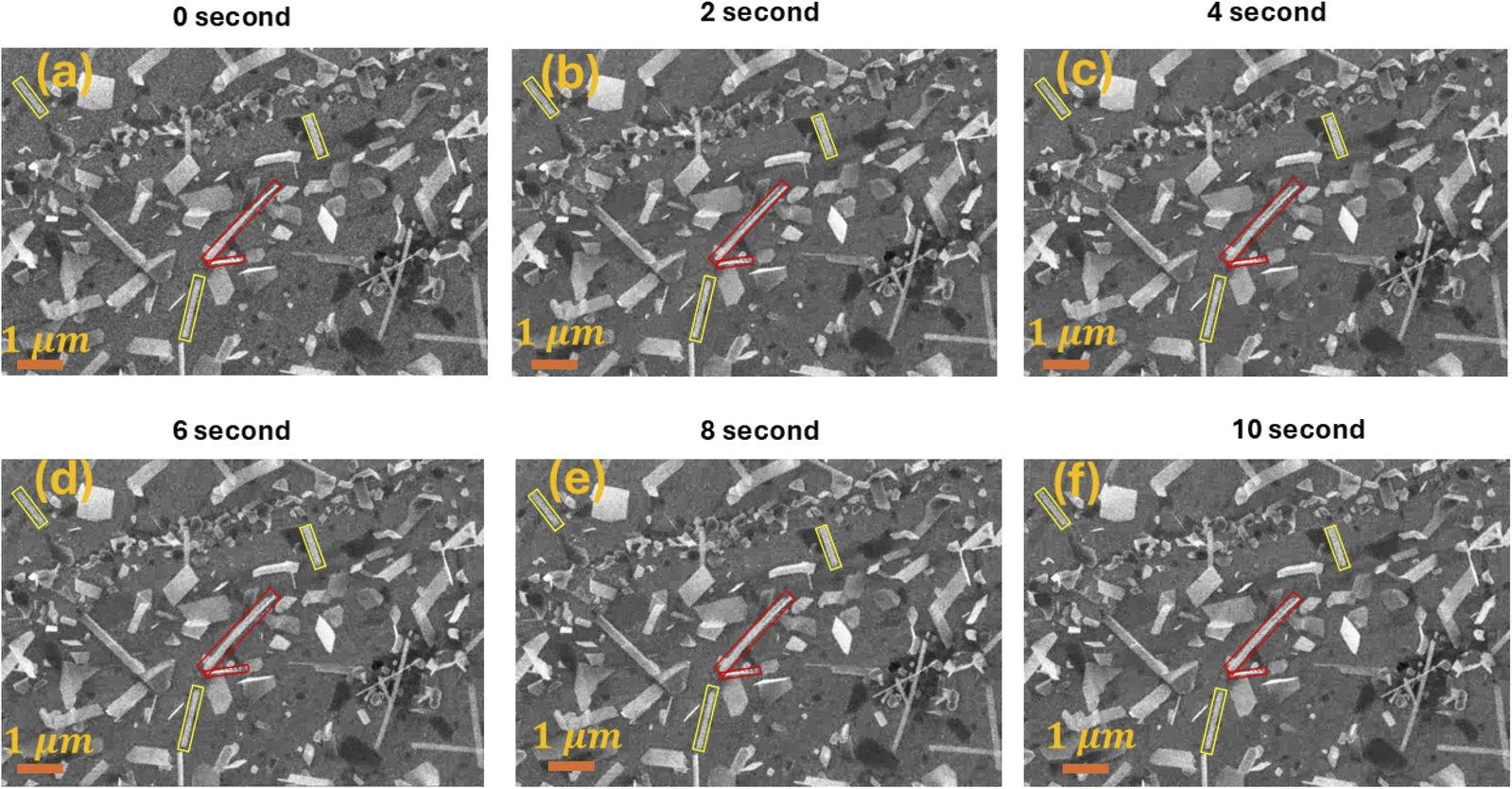
Control experiment for distinguishing mechanical motion from SEM imaging artifacts. (a–f) FESEM images acquired during continuous electron-beam exposure at 0, 2, 4, 6, 8, and 10 s respectively. The nanowire marked by the red box exhibits progressive edge displacement/broadening toward its free end, whereas the nanowire regions marked by yellow boxes serve as non-vibrating references and remain essentially unchanged under the same exposure conditions.

Fig. 5(a) shows SEM images of a cantilevered Bi_2_Te_3_ nanowire 3 (NW3) under continuous electron-beam irradiation along with the corresponding grey-scale intensity profiles extracted across the wire. In contrast to Fig. 2 and 3, the intensity distribution at the tip is no longer well described by a single Gaussian. Instead, the profiles exhibit pronounced peaks near the extremities with a finite intensity in the central region. This deviation from a Gaussian distribution indicates that the motion is no longer purely thermally driven but is dominated by externally driven oscillations.

**Fig. 5:**
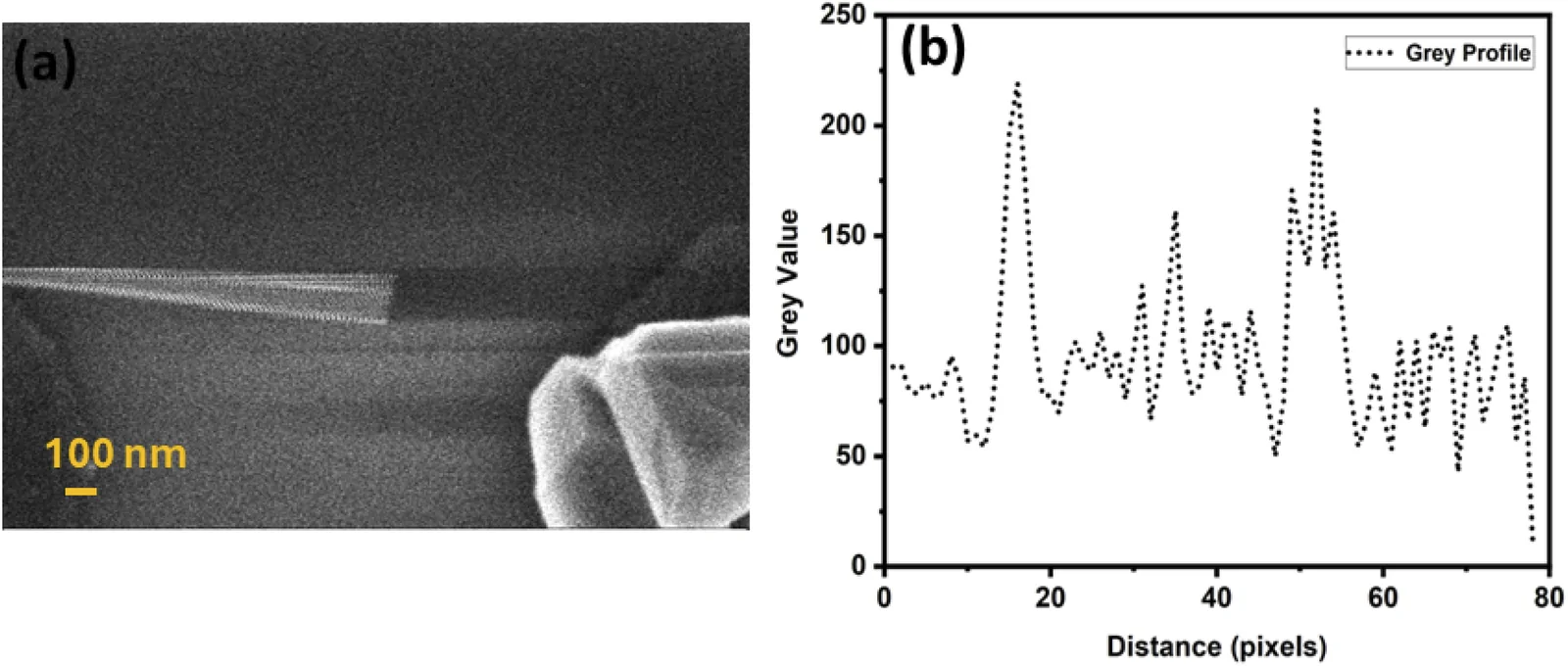
(a) Representative SEM images of a cantilevered Bi_2_Te_3_ nanowire (NW3) under electron beam irradiation. (b) Corresponding grey-scale intensity profiles.

In the present experiment, beam-induced charging is treated as the physical excitation mechanism responsible for the observed electrostatic actuation rather than as a purely imaging-induced broadening mechanism. Under electron-beam exposure, the nanowire accumulates charge due to an imbalance between incident and emitted electrons. The electrostatic force generated by the accumulated charge can be approximated as

8 *F*_e_(*t*) *α V*(*t*)^2^

which acts as a driving term in the equation of motion of the cantilever. In this forced regime, the nanowire act as a driven oscillator rather than fluctuating solely due to thermal energy, during this oscillatory motion a frequency component of the driving force overlap with a natural frequency of the cantilever which leads to the large amplitude oscillation. We can understand the corresponding intensity profile in Fig. 5(b) in terms of probability distribution of a driven oscillator as follows. For a harmonic oscillation, *y*(*t*) = *A* sin (*ωt*) thus the probability density of finding the oscillator at position *y* is given by9
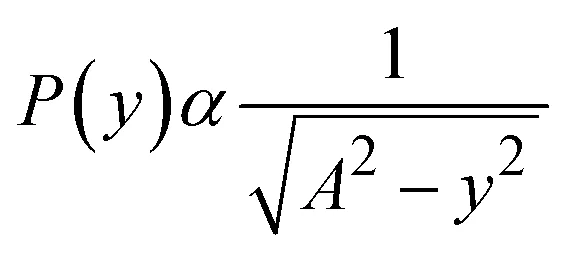
which diverges near the turning points (*y* = ±*A*). This results in higher intensity near the extremities and reduced intensity at the center, consistent with the profiles observed experimentally. The presence of a finite central hump, indicates that the motion is not purely single-mode harmonic but involves contributions from multiple modes.

The spatial dependence of the displacement can be described using the mode shape functions using eqn (2). The fundamental mode (*n* = 1) produces a monotonic increase in amplitude along the length while higher-order modes introduce additional curvature and local maxima. The experimental profile deviates from a simple gaussian form, with the central features suggesting that the oscillation is a superposition of fundamental and higher modes. The presence of higher modes in oscillation reflects the role of electron beam as a broadband excitations rather than a simple force driver. Thus, Fig. 5 demonstrates the transition from thermally dominated motion to driven oscillations in Bi_2_Te_3_ nanowires. The non-Gaussian intensity profiles, together with the features associated with higher modes, are consistent with electron-beam-induced charging contributing substantially to the observed dynamics. This provides evidence that SEM-based electron-beam excitation can access vibrational behaviour beyond the fundamental mode and can be used to probe the full modal structure of nanoscale cantilevers.

Fig. 6 shows the extraction of the vibration amplitude along the length of the nanowire and its comparison with theoretical mode shapes. The nanowire (Fig. 6(a)) edges were first identified using edge detection, and a centreline was constructed (Fig. 6(b)). The local amplitude was then obtained as the distance between the centreline and the edge at different positions along the length. This provides a direct measure of the spatial variation of displacement, *A*(*x*), along the cantilever.

**Fig. 6:**
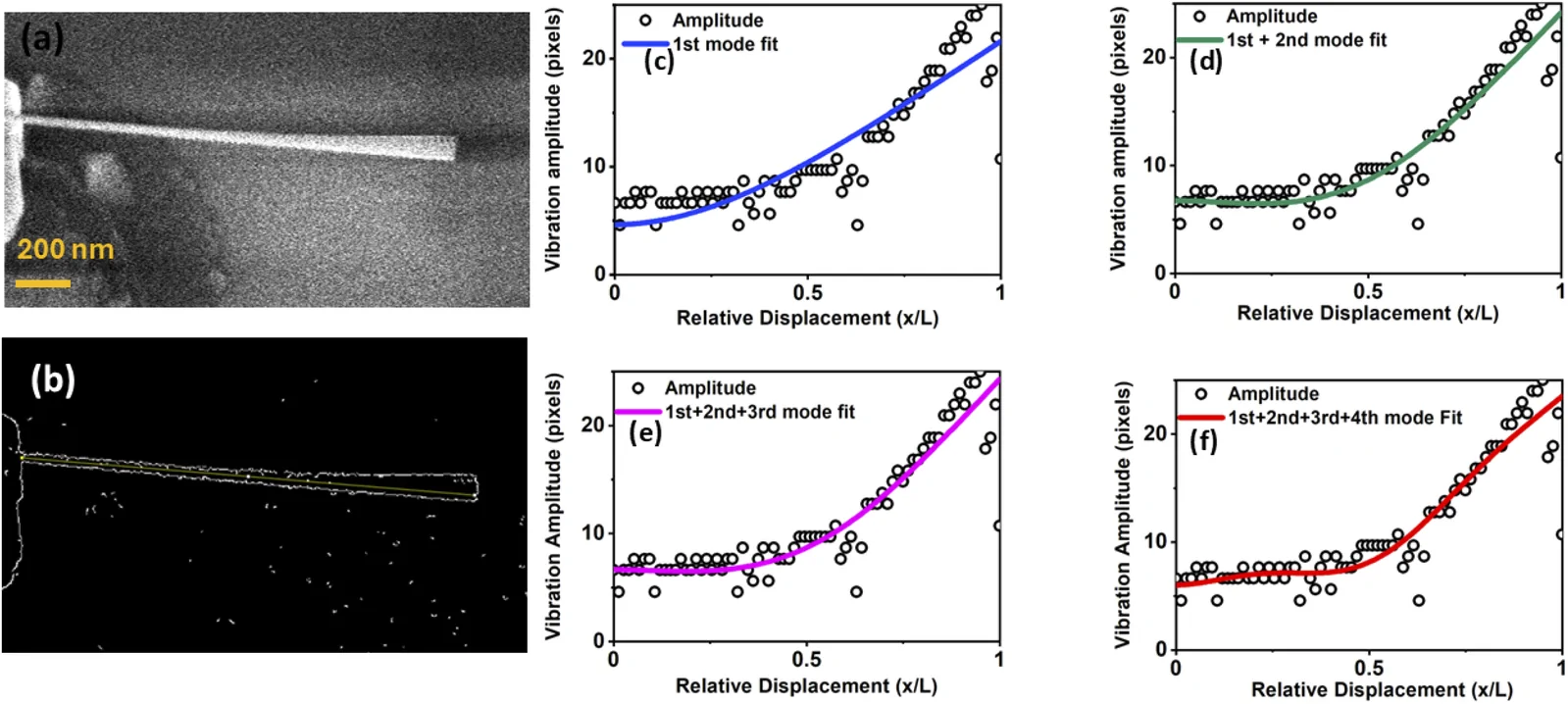
Extraction and fitting of the vibration amplitude profile along the nanowire length (NW3). (a and b) Edge detection and centreline identification using the Canny algorithm. (c–f) Experimental amplitude data fitted with theoretical mode shapes: fundamental mode only, and superposition of higher-order modes.

For a cantilever beam, the displacement profile is expected to follow the mode shape functions obtained from the Euler–Bernoulli equation. The general form of the mode shape is given by eqn (2) with characteristic solution at *n* = 1 yield fundamental mode *β*_1_*L* ≈ 1.875.

The experimentally extracted amplitude profile is first fitted using only the fundamental mode (Fig. 6(c)). While this captures the overall trend of increasing displacement toward the free end, a clear deviation is observed, particularly in the mid-section of the nanowire. The experimental data show a slight curvature and enhancement that cannot be accounted for by a single-mode description.

To account for this, the displacement is expressed as a superposition of modes,

10 *A*(*x*) = *a*_1_*Y*_1_(*x*) + *a*_2_*Y*_2_(*x*)

where *Y*_1_(*x*) and *Y*_2_(*x*) correspond to the fundamental (*n* = 1) and first (*n* = 2) overtone with characteristic solution *β*_2_*L* ≈ 4.694 (*n* = 2),respectively up to *n* = 4 (Fig. 6(d–f)). The existence of higher modes places Bi_2_Te_3_ nanowire cantilever in the nanoelectromechanical systems (NEMS) range.^24^ Addition of the 2nd mode to the experimental data improves the fitting with reproducing overall shape as well as the local deviation observed in profile. The improved agreement obtained by including the second mode indicates that the observed oscillation contains contributions from multiple vibrational modes. Such behaviour is consistent with the driving mechanism discussed earlier, where electron-beam-induced charging provides a broadband excitation. Unlike harmonic driving at a fixed frequency, this type of excitation can couple to multiple eigenfrequencies of the system, leading to a superposition of mode shapes. Thus, Fig. 6 indicates that the observed vibration under electron-beam excitation contains contributions from multiple vibrational modes. The ability to resolve and fit higher-order mode contributions is consistent with classical beam dynamics and highlights the role of the excitation mechanism in determining the observed motion.

Electron-beam-induced excitation and oscillatory motion of nanowires and nanoresonators have been reported previously, including studies in which electron-beam charging provides the driving force for mechanical oscillations, as well as *in situ* electron-microscopy measurements of nanomechanical motion.^8–12^ The present observations are consistent with this established electromechanical picture. Our contribution is therefore not the introduction of a new electron-beam excitation mechanism, but the application of this established contactless excitation approach to cantilevered Bi_2_Te_3_ nanowires together with spatial profile analysis, multimode analysis, and temporal charging-relaxation measurements within the same SEM platform. The results provide a systematic characterization of the electron-beam-induced electromechanical response of Bi_2_Te_3_ nanowire cantilevers.

Fig. 7 shows the time evolution of the nanowire 4 (NW 4) deflection under continuous electron-beam exposure. The FESEM images in (Fig. 7(a–c)) show progressive bending as the irradiation time increases from 0 to 60 s, followed by saturation. Fig. 7(d) shows the corresponding tip deflection as a function of irradiation time, while Fig. 7(e and f) show the spatial deflection profile and its comparison with the fundamental and higher-order cantilever modes.

**Fig. 7:**
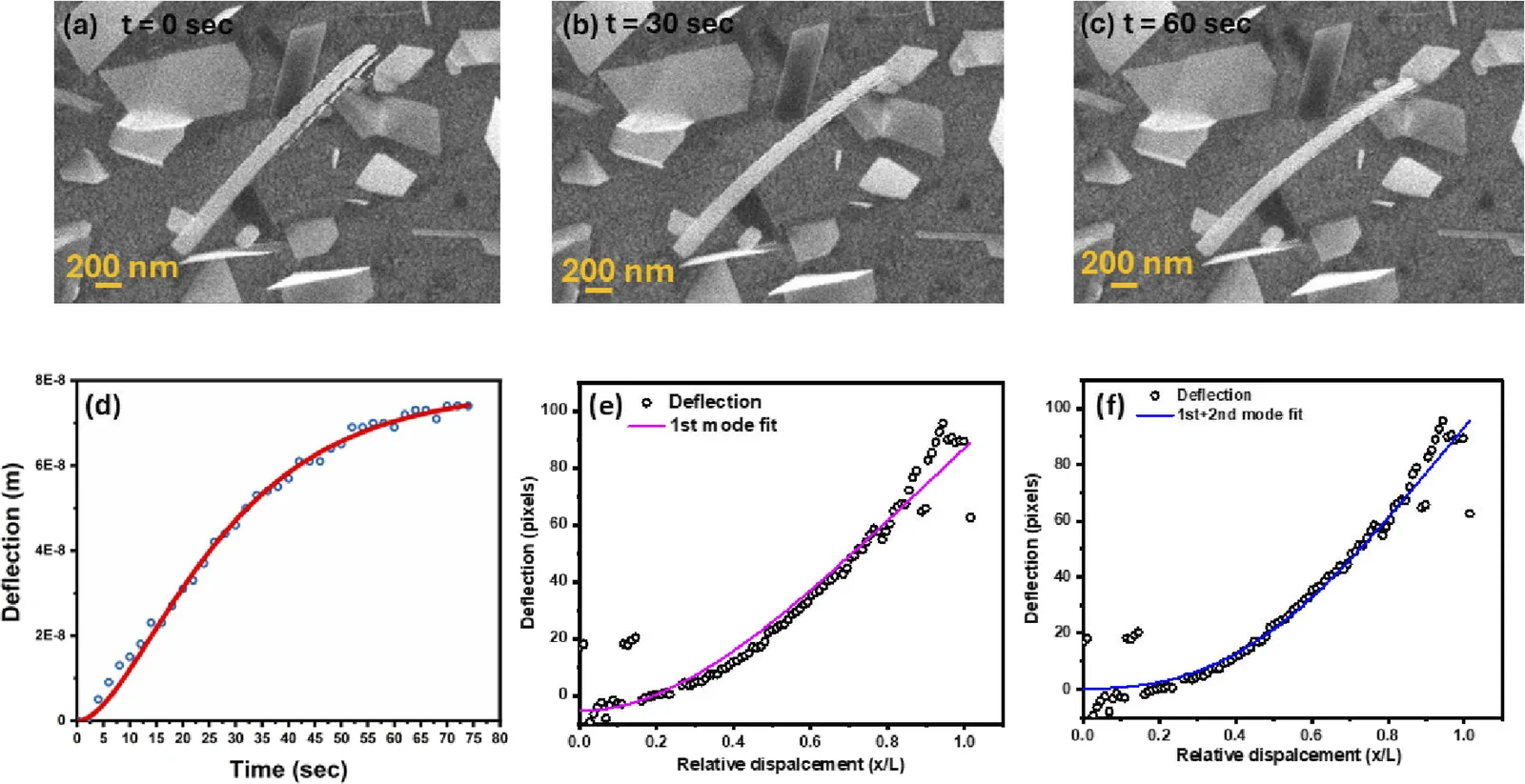
Time evolution of nanowire (NW4) deflection under continuous electron beam exposure. (a–c) show the position of the Cantilever at *t* = 0, *t* = 30 s & *t* = 60 s respectively. We can observe progressive bending at different time intervals. (d) shows the measured tip deflection as a function of irradiation time, fitted using the charging-induced deformation model described by eqn (13). (e and f) Experimental deflection data fitted with theoretical mode shapes: fundamental mode only, and superposition of higher-order modes.

This behaviour can be understood in terms of electron-beam-induced charging of the nanowire. The incident primary electron current *I*_p_ is partially balanced by secondary and backscattered electron emission, resulting in a net charging current given by,^25^

11 *I*_net_ = *I*_p_ − (*I*_se_ + *I*_bse_) = *I*_p_(1 − *δ*)

where *I*_p_ is the primary beam current and *δ* is the total emission yield. The accumulated charge leads to a time-dependent surface potential *V*(*t*), which evolves according to12
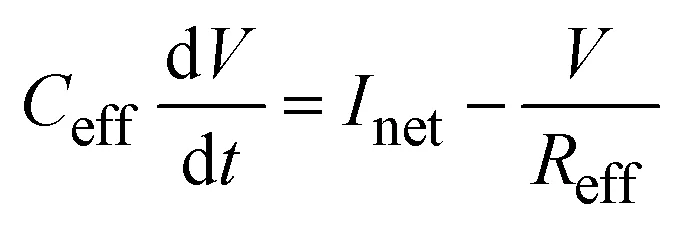
Here, *C*_eff_ and *R*_eff_ represent effective parameters describing the aggregate charging and leakage response of the irradiated nanowire/substrate system rather than spatially resolved electrical elements. Because the experimentally measured quantity is the global tip deflection as a function of irradiation time rather than a spatially resolved electrical potential, the lumped *R*_eff_*C*_eff_ representation is used here as an effective description of the dominant charging-relaxation timescale of the coupled nanowire/substrate system. A more general distributed-RC description could account for spatially varying resistance and capacitance along the nanowire; however, such spatial electrical information is not directly resolved by the present contactless SEM measurement. The solution of the eqn (12) gives an exponential approach to a steady-state voltage,13
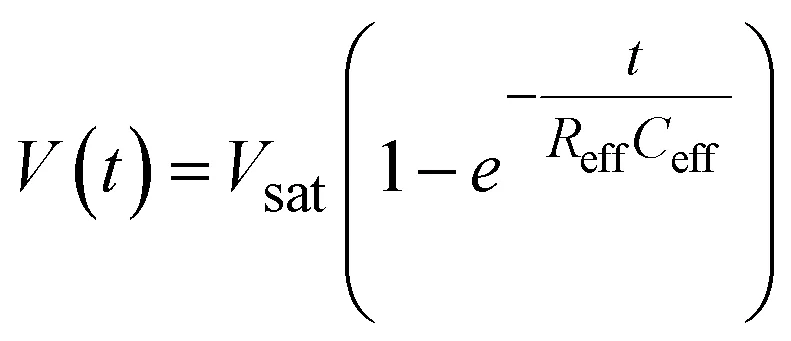
where *V*_sat_ = *I*_net_*R* and *τ*_eff_ = *R*_eff_*C*_eff_ is the characteristic charging time constant, which implies that the electrostatic force acting on the nanowire increases with time and eventually saturates, can be approximated as:

14 *F*_e_(*t*) *α d*(*t*) *α V*(*t*)^2^

Using the voltage evolution above, the quasi-static displacement therefore follows *d*(*t*) ∝ [1 − exp(−*t*/*τ*)]^2^, with the proportionality factor determined by the effective electrostatic coupling and mechanical stiffness. The experimentally fitted relaxation time consequently characterizes the temporal charging-leakage response that drives the observed deformation.

Here *d*(*t*) corresponds to deflection profile with time evolution, fitting eqn (13) to the measured tip-deflection data in Fig. 7(d) yields an effective relaxation time of *τ*_eff_ = 19.96 ± 0.27 s and a final tip deflection of approximately 8.8 nm. The time-dependent increase in deflection is consistent with the gradual development of electrostatic force during charge accumulation and its subsequent saturation as leakage balances charging. The uncertainty of ±0.27 s represents the uncertainty associated with the temporal fit and should not be interpreted as a repeated-measurement reproducibility interval, since independent repeated measurements under identical conditions were not performed in the present dataset. The measured relaxation time, *τ* = 19.96 ± 0.27 s, is interpreted as an effective charging-relaxation timescale of the irradiated nanowire/substrate system. Electron-beam-induced charge accumulation followed by leakage provides the primary mechanism for the observed quasi-static bending. Native surface oxide, surface contamination are negligible due to the ultra high vacuum curing of samples,^25–29^ defect- or trap-mediated charge storage, bulk conduction, and substrate-assisted leakage may also contribute minor to the effective relaxation and cannot be independently separated using the present contactless SEM measurement. The saturation of the tip deflection at longer irradiation times is consistent with the balance between charge accumulation and leakage through the substrate.^30^

The observed bending also suggests that, in addition to elastic deformation, there may be contributions from time-dependent processes such as creep or plastic deformation, especially under prolonged exposure where local heating and stress are present. However, the initial exponential behaviour is well captured by the charging model, indicating that electrostatic effects dominate the early-time response. Thus, Fig. 7 demonstrates that irradiation due to electron beam induces dynamic oscillations along with the time dependent deformation due to charge accumulation. In this way the electron beam plays a vital role as dynamic excitation source and quasi static actuator. This dual nature of electron beam highlights the cruciality of the determination of mechanical behavior of nanowire under FESEM conditions by electrostatic effects.

The four nanowires provide independent measurements across different device geometries. Because their lengths and radii are not identical, the spread in the extracted Young's modulus values is treated as descriptive cross-device variation rather than as a strict measure of reproducibility for a fixed geometry. Across the four measured nanowires, the extracted Young's modulus values range from 12.56 to 28.6 GPa, with a descriptive mean of 20.93 GPa, a sample standard deviation of 6.65 GPa, and a coefficient of variation of approximately 31.67%. Because the nanowires have different lengths and radii, this variation is not treated as a confidence interval for a single fixed geometry, but as a descriptive measure of cross-device variation.

In the Table 2, stiffness (spring constant, *k*) is calculated by 
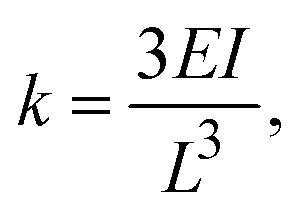
 where *E* is young's modulus, *I* is area moment of inertia and rigidity is given by *EI*. The measured parameters provide a comparison of the mechanical response across the investigated Bi_2_Te_3_ nanowires. The Young's moduli of ∼12.56–28.6 GPa are in valid limit and in close approximation with previously reported value for nanostructures and well below the bulk and single crystal limit. The observed values of young's modulus in studied nanowire system further suggests surface softening condition as discussed in diameter-dependent softening arise from Bi_2_Te_3_'s quintuple-layer structure and GaN nanowires,^31–34^ the same structural anisotropy responsible for low thermal conductivity and high thermoelectric ZT.^35–38^ The measured RC time constant, *τ* = 19.96 ± 0.27 s, is interpreted as an effective charging-relaxation timescale of the electron beam irradiated nanowire/substrate system.

**Table 2:** Physical parameters; Youngs modulus (*E*), stiffness (*k*) and rigidity of Bi_2_Te_3_ nanowires

Nanowire	∼*L* (nm)	∼*R* (nm)	*E* (GPa)	Stiffness (*k*) (N m^−1^)	Rigidity (N m^2^)
NW1	5300	23.5	22.6	1.09 × 10^−4^	5.41 × 10^−21^
NW2	1480	10.9	12.56	1.29 × 10^−4^	1.39 × 10^−22^
NW3	1700	18	19.95	1.00 × 10^−3^	1.64 × 10^−21^
NW4	2300	76	28.6	1.85 × 10^−1^	7.49 × 10^−19^

## Conclusion

In summary, we have studied the electro-thermo-mechanical response of cantilevered Bi_2_Te_3_ nanowires under *in situ* electron-beam excitation. For NW1 and NW2, thermally driven intensity profiles yield Young's moduli of 22.6 GPa and 12.56 GPa, consistent with reported mechanical properties of Bi_2_Te_3_. For NW3, further surface softening model confirms Young's modulus of 19.95 GPa, while the multimode analysis indicates broadband electron-beam excitation in the nanoscale electromechanical regime. For NW4, we obtain a Young's modulus of 28.6 GPa, and the time-dependent bending is described by an effective charging-relaxation time constant of *τ* = 19.96 ± 0.27 s, consistent with electron-beam-induced charge accumulation and leakage. Although Bi_2_Te_3_ is a well-established topological-insulator material and previous transport studies have reported surface-state contributions, the present measurements do not directly resolve electronic transport. The electromechanical response is therefore interpreted primarily in terms of electron-beam-induced charging and electrostatic actuation, while a minor contribution from surface-state-mediated charge redistribution remains to be established by direct electronic measurements. These results illustrate the use of electron-beam excitation as a contactless approach for probing and driving electromechanical dynamics in Bi_2_Te_3_ nanowires.

## Author contributions

Abhinav Srivastav: methodology, formal data analysis, investigation, software, writing original draft. Priyank Tripathi: methodology, formal data analysis, investigation, software, reviewed, writing final draft. Reena Yadav: sample preparation, characterization, resources, formal analysis. Mandeep Kaur: methodology, sample preparation, resources, characterization, formal analysis. Sahil Verma: data curation and resources. Ravita: data curation and resources. Sudhir Husale: supervision, conceptualization, methodology, FIB – nanodevice fabrication, formal data analysis, software, investigation, validation, writing original draft, writing – review and editing final draft, resources.

## Conflicts of interest

The authors declares no conflict of interest.

## Supplementary Material

NA-OLF-D6NA00841K-s001

NA-OLF-D6NA00841K-s002

NA-OLF-D6NA00841K-s003

NA-OLF-D6NA00841K-s004

NA-OLF-D6NA00841K-s005

NA-OLF-D6NA00841K-s006

NA-OLF-D6NA00841K-s007

NA-OLF-D6NA00841K-s008

NA-OLF-D6NA00841K-s009

NA-OLF-D6NA00841K-s010

## Data Availability

The raw experimental data supporting the findings of this study are available in the supplementary information (SI) accompanying this article. Supplementary information: raw experimental datasets in Microsoft Excel format and video files demonstrating the response of Bi_2_Te_3_ nanowires under electron-beam excitation. See DOI: https://doi.org/10.1039/d6na00841k.
