# Supplementary material for: Coupled electro-mechanical response of Bi2Te3 nanowires probed by electron-beam excitation
Source: Nanoscale Adv. 2026 Oct 26. Online ahead of print. doi: 10.1039/d6na00841k (PMC13726101; doi:10.1039/d6na00841k)
Supplement: Supplementary file 1 [file NA-OLF-D6NA00841K-s009.pdf]

Please do not adjust margins

Supplementary Information (SI) for Nanoscale Advances.
This journal is © The Royal Society of Chemistry

PAPER

Supplementary material

Coupled Electro-Mechanical Response of Bi₂Te₃ Nanowires Probed by Electron-Beam Excitation

Abhinav Srivastav[b,c,1†], Priyank Tripathi[a,b,1†], Reena Yadav[a,b], Sahil Verma[a,b], Ravita[a,b], Mandeep Kaur[b] & Sudhir Husale[a,b*]

Experimental Method:

Bi₂Te₃ nanowires used in this study were mechanically exfoliated from bulk crystals and transferred onto conductive substrates suitable for electron microscopy. The samples were inspected using a field-emission scanning electron microscope (FESEM), where individual nanowires protruding from the substrate edge or loosely anchored at one end were identified and selected for further measurements[1-3]. Particular care was taken to choose nanowires with a well-defined cantilever geometry, where one end remained clamped to the substrate while the other end was free to oscillate.

All measurements were carried out inside the SEM chamber under high vacuum conditions. The electron beam was operated at accelerating voltages in the range of 5–15 kV with beam currents adjusted to ensure stable imaging while minimizing beam-induced damage[4,5]. Under continuous electron beam exposure, selected nanowires exhibited visible oscillatory motion, which was recorded in real time using the SEM imaging system. The imaging conditions (scan rate, magnification, and working distance) were optimized to clearly resolve the nanowire profile and its temporal broadening during vibration[6,7].

Data Analysis :

To quantify the vibrational amplitude, intensity profiles were extracted from SEM images along directions perpendicular to the nanowire axis. The nanowire was oriented approximately horizontal in the field of view to facilitate analysis. Grey-scale intensity profiles were obtained at both the clamped base and the free end of the nanowire. The base region, where no motion is expected, was used to determine the intrinsic instrumental broadening. The profile at the free end showed additional broadening due to vibrational motion. Both profiles were fitted using Gaussian functions, and the variance associated with the vibrational contribution was obtained from the difference between the tip and base widths.

Image Processing :

[a.] *Academy of Scientific and industrial Research (AcSIR), Ghaziabad-201002, India.*
[b.] *Council of Scientific and Industrial Research, National Physical Laboratory, Dr. K.S. Krishnan Marg, New Delhi – 110012, India.*
[c.] *Department of Physics, Indian Institute of Technology Delhi, Hauz Khas, New Delhi 110016, India.*
[d.] ** Correspondence Email : husalesc.nplindia@csir.res.in*

[†] ***Abhinav Srivastav and Priyank Tripathi equally contributed to this work.***

Please do not adjust margins

In order to analyse the spatial distribution of vibration along the nanowire, edge detection was performed on SEM images using a standard Canny algorithm[8-10]. The upper and lower edges of the nanowire were identified, and a centreline was constructed along its length. The local amplitude was then estimated from

the distance between the centreline and the edges at different positions. These data were compared with analytical mode shapes for a cantilever beam to identify the contribution of fundamental and higher-order modes.

**Bi₂Te₃ type**	**Method**	**Young's modulus**	**Reference**
Bi₂Te₃ 2D nanosheets, (5–14 nm)	AFM nanoindentation	11.6–24.9 GPa	[11]
Bulk Bi₂Te₃	Nanoindentation / bulk mechanical comparison	41.80 GPa	[12]
Sputtered Bi₂Te₃ thin films	Nanoindentation	52.31–58.10 GPa	
Single-crystal Bi₂Te₃ topological insulator	Depth-sensing nanoindentation	32.4 ± 2.9 GPa	[13]
Bi₂Te₃ nanowire cantilevers	SEM thermal-vibration broadening	12.56-28.6 GPa	Present work

Table S1 : Comparative table of reported Young's modulus of different Bi_2Te_3 derived systems.

Statistical analysis of length scales of nanowires-

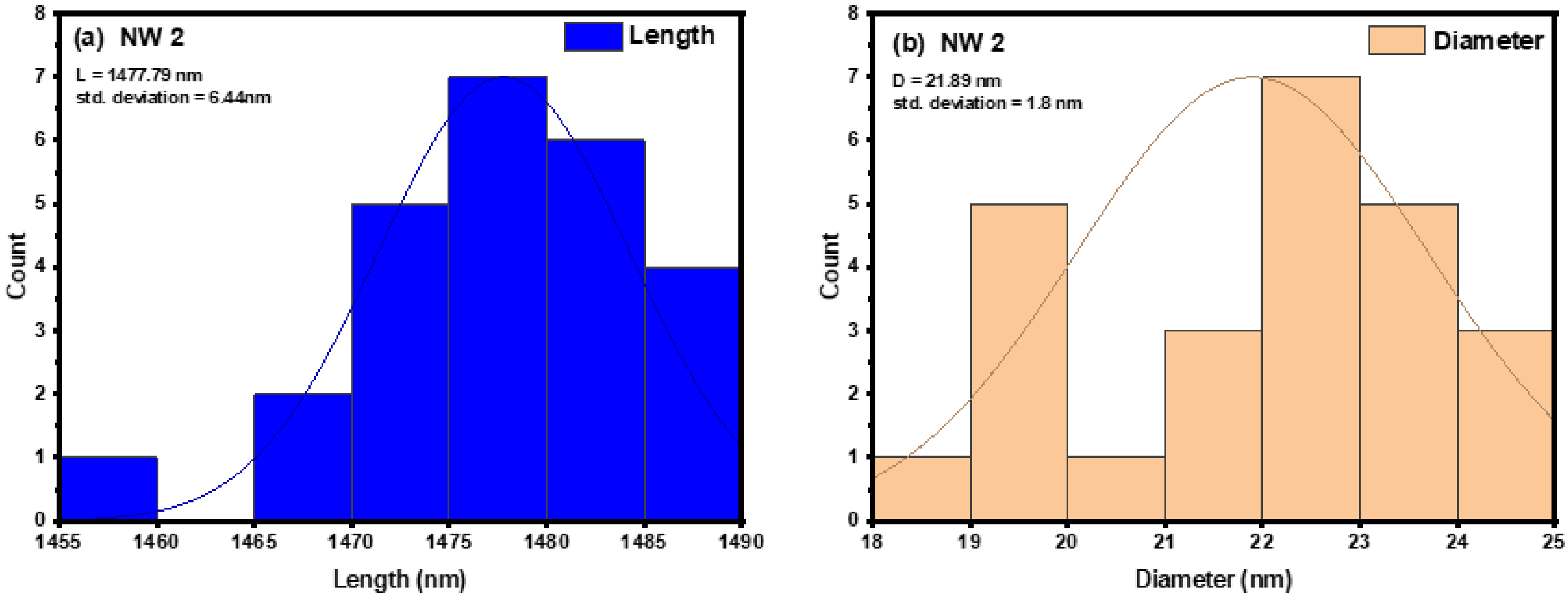

Figure S1. *Statistical analysis of the length and diameter distributions of the investigated Bi₂Te₃ nanowire. Histograms with Gaussian fits showing the distributions of (a) length and (b) diameter for NW2. The corresponding mean values and standard deviations are indicated in each panel.*

Please do not adjust margins

Uncertainty calculation-

Measured uncertainty from image profile. Uncertainty in temperature is fixed at 2 K.

nanowire	$\square_{tip}$	$\square_{tip}$	$\square_{base}$	$\square_{base}$	$\square_{vib}$	$\square_{vib}$	L	$\square_L$	R	$\square_R$	T (K)	$\square_T$ (K)
NW2	11.38	0.84	9.88	0.6	5.64	1.98	1478	6.44	10.9	0.9	298	2

Table S2 : *Measured uncertainty from image profile of NW2. Uncertainty in temperature is fixed at 2 K.*

Here All length scales ($\square_{tip}$,$\square_{tip}$,$\square_{base}$,$\square_{base}$,$\square_{vib}$,$\square_{vib}$, L,$\square_L$,R,$\square_R$) are in nm.

Here, $\square_{vib} = \sqrt{\square_{tip}^2 - \square_{base}^2}$ and uncertainty in $\square_{vib}$ is given by $\square_{vib}$ as follows[14]:

$$\square_{vib} = \sqrt{\left(\frac{\square_{base}}{\square_{vib}}\square_{base}\right)^2 + \left(\frac{\square_{tip}}{\square_{tip}}\square_{tip}\right)^2} \qquad (S1)$$

Finally, the uncertainty in the vibrational width was propagated to Young's modulus using the relation[15]

$$E = \frac{4k_B T L^3}{3\square r^4 \square_{vib}^2} \qquad (S2)$$

Giving[14]

$$\left(\frac{u_E}{E}\right)^2 = \left(\frac{u_T}{T}\right)^2 + \left(3\frac{u_L}{L}\right)^2 + \left(4\frac{u_r}{r}\right)^2 + \left(2\frac{u_{\square vib}}{\square_{vib}}\right)^2 \qquad (S3)$$

Thus, the uncertainties in temperature, nanowire length, radius and extracted vibrational width are propagated consistently into the final mechanical-property determination.

Uncertainty	NW2
$\frac{\square_T}{T}$	0.67%
$3\square_L/L$	1.31%

Please do not adjust margins

$4\square_{R}/R$	33.03%
$^{2}\square_{vib}/\square_{vib}$	70.21%
$\square_{E}/E$	77.61%
$\square_{E}$	9.75 GPa

Table S3 *: Individual contributions of the measured parameters to the propagated uncertainty in Young's modulus. The uncertainty contributions are expressed as fractional terms obtained from the first-order propagation of* E*. The combined uncertainty is calculated by quadrature assuming independent input uncertainties.*

The uncertainty budget indicates that the dominant contribution to the final Young's modulus uncertainty arises from the uncertainty in the extracted vibrational width, owing to the subtraction of the tip and base variances. The radius uncertainty provides the next significant contribution because of the R^{-4} dependence of Young's modulus, whereas the contributions from temperature and nanowire length are comparatively small.

References :

1. Sharma, A., Srivastava, A.K., Senguttuvan, T.D. and Husale, S., 2017. Robust broad spectral photodetection (UV-NIR) and ultra high responsivity investigated in nanosheets and nanowires of Bi2Te3 under harsh nano-milling conditions. *Scientific reports*, *7*(1), p.17911.
2. Yadav, R., Bhattacharyya, B., Pandey, A., Kaur, M., Aloysius, R.P., Gupta, A. and Husale, S., 2021. Accessing topological surface states and negative MR in sculpted nanowires of Bi2Te3 at ultra-low temperature. *Journal of Physics: Condensed Matter*, *33*(8), p.085301.
3. Bhattacharyya, B., Singh, B., Aloysius, R.P., Yadav, R., Su, C., Lin, H., Auluck, S., Gupta, A., Senguttuvan, T.D. and Husale, S., 2019. Spin-dependent scattering induced negative magnetoresistance in topological insulator Bi2Te3 nanowires. *Scientific Reports*, *9*(1), p.7836.
4. Goldstein, J.I., Newbury, D.E., Michael, J.R., Ritchie, N.W., Scott, J.H.J. and Joy, D.C., 2017. *Scanning electron microscopy and X-ray microanalysis*. springer.
5. Tripathi, P., Nayak, D., Kaur, M., Verma, S., Senapati, K. and Husale, S., 2026. Geometry-driven dimensional crossover from weak localization to superconducting fluctuations in nanostructured NbN networks. *Nanoscale*, 2026, **18**, 13958 – 13968.
6. Reimer, L., 2000. Scanning electron microscopy: physics of image formation and microanalysis. *Measurement Science and Technology*, *11*(12), pp.1826-1826.
7. Reimer, L. and Microscopy, S.E., 1985. Physics of image formation and microanalysis. *Springer*, *45*, p.135.
8. Rong, W., Li, Z., Zhang, W. and Sun, L., 2014, August. An improved CANNY edge detection algorithm. In *2014 IEEE international conference on mechatronics and automation* (pp. 577-582). IEEE.

Please do not adjust margins

9. Zhao, H., Qin, G. and Wang, X., 2010, October. Improvement of canny algorithm based on pavement edge detection. In *2010 3rd international congress on image and signal processing* (Vol. 2, pp. 964-967). IEEE.
10. Kumar, M. and Saxena, R., 2013. Algorithm and technique on various edge detection: A survey. *Signal & Image Processing*, *4*(3), p.65.
11. Guo, L., Yan, H., Moore, Q., Buettner, M., Song, J., Li, L., Araujo, P.T. and Wang, H.T., 2015. Elastic properties of van der Waals epitaxy grown bismuth telluride 2D nanosheets. *Nanoscale*, *7*(28), pp.11915-11921.
12. Tasi, C.H., Tseng, Y.C., Jian, S.R., Liao, Y.Y., Lin, C.M., Yang, P.F., Chen, D.L., Chen, H.J., Luo, C.W. and Juang, J.Y., 2015. Nanomechanical properties of Bi2Te3 thin films by nanoindentation. *Journal of Alloys and Compounds*, *619*, pp.834-838.
13. Lamuta, C., Cupolillo, A., Politano, A., Aliev, Z.S., Babanly, M.B., Chulkov, E.V., Alfano, M. and Pagnotta, L. (2016), Nanoindentation of single-crystal Bi_2Te_3 topological insulators grown with the Bridgman–Stockbarger method. Phys. Status Solidi B, 253: 1082-1086.
14. Taylor, B.N. and Kuyatt, C.E., 1994. *Guidelines for evaluating and expressing the uncertainty of NIST measurement results* (Vol. 1297). Gaithersburg, MD: US Department of Commerce, Technology Administration, National Institute of Standards and Technology.
15. Nonaka, K., Tamaru, K., Nagase, M., Yamaguchi, H., Warisawa, S.I. and Ishihara, S., 2009. Evaluation of thermal–mechanical vibration amplitude and mechanical properties of carbon nanopillars using scanning electron microscopy. *Japanese journal of applied physics*, *48*(6S), p.06FG07.

This journal is © The Royal Society of Chemistry 20xx

Please do not adjust margins
